# IoT–Blockchain: Harnessing the Power of Internet of Thing and Blockchain for Smart Supply Chain

**DOI:** 10.3390/s21186048

**Published:** 2021-09-09

**Authors:** Neda Azizi, Heliyeh Malekzadeh, Peyman Akhavan, Omid Haass, Shahrzad Saremi, Seyedali Mirjalili

**Affiliations:** 1 Torrens University Australia, Melbourne, VIC 3000, Australia; neda.azizi@torrens.edu.au (N.A.); shazi.saremi@torrens.edu.au (S.S.); 2 Industrial Engineering Department, Iran University of Science and Technology, Tehran 16844, Iran; helymz@yahoo.com (H.M.); akhavan@mut.ac.ir (P.A.); 3 Iran Knowledge Management Association (IKMA), Tehran 16788, Iran; 4 School of Property, Construction and Project Management, RMIT University, Melbourne, VIC 3000, Australia; omid.haass@rmit.edu.au; 5 Yonsei Frontier Lab, Yonsei University, Seoul 03722, Korea

**Keywords:** smart supply chain management (SSCM), internet of things (IoT), blockchain (BC), Dematel method, Delphi method

## Abstract

This study aims to build smart supply chains for the first time using the internet of things (IoT) and blockchain. Classification and clarification of causal relationships can provide a useful framework for researchers and professionals who seek to implement an intelligent supply chain using IoT tools in a blockchain platform, and it also demonstrates the intensity of communications indicating such relationships. The research methodology is mixed method, comprised of qualitative and quantitative methods. The qualitative method includes the Delphi method used for selecting indigenous components and features proper for the pattern. The quantitative method is the Dematel method used for assessing the relationships between the available concepts in the pattern and accessing the network structure between components. Interpretative Structural Modeling is also employed to classify the network structure obtained from the Dematel technique. The findings of the study identify indicators of IoT and blockchain as causes based on Dematel, application of tools, components interconnectedness, optimal decision making, automatedness, integration, innovation and learning, which are indicators of smart supply chain, are the effects in this study.

## 1. Introduction 

Smart supply chain management is concerned with providing the right goods at the right time with the right amount at the right place at the right price under the right conditions for the right customer [1].

In the new era, global supply chains are more complex and are confronted with frequent uncertainty [2] and tough competitions in the market [3]. Moreover, one of the major concerns of enterprises is to collect their correct and high-quality data in the best time possible, as fast as possible, to provide it to their internal and foreign sectors easily, and to have control over the data and their sharing so as to make optimal large-scale decisions. For this purpose, it is important to find a useful instrument for data production, security, sharing and the ability of real-time analysis. Therefore, we need to create a smart supply chain in this era so that we can address enterprises’ needs and collect the relevant data first, and then analyze the data for deeper and more complete understanding of the current performance [4].

There are different expenditures on a supply chain, which can be eliminated, for example, the expenses related to defective products [5]. The source of failure is identified and the damaged products are strategically removed, and this way the shutting down of the production line is prevented [5,6]. The smart supply chain management is facilitated by identification, measurement and pursuing key processes with IoT.

Moreover, by elimination of formal procedures, the costs are reduced and the processes are accelerated. Further, if we know that the chain members cannot use low-quality or fake products, and we ensure the originality and quality of products, we still save in the expenses. This needs transparency, visibility and traceability of products by eliminating the intermediaries, which requires use of unchangeable data, distributed reservoirs and controlled access, for which strategies such as use of blockchain platform may be useful.

One other problem faced in a traditional supply chain is the lack of trust between suppliers and their identity verification problems. This may be solved by making supply chains smart, using smart contracts and recording the data in a reliable and unchangeable database with controlled accessibility [7,8].

Thus, every supply chain is growing and developing rapidly, and management of assets in all global supply chains requires a novel approach. Wi-Fi, RFIDs, and GSM/GPS, for example, may have their own area of specialty [9,10], but they are not designed in such a way that they can easily and securely connect a huge number of IoT devices. Complex cases, increased energy costs, globalization, safety threats and regulatory pressures have made supply chain managers adopt and adapt solutions, which may provide them with a better understanding of their performance in a larger economy, environment and society.

Huckle et al. [11] pointed out that energy costs [12], globalization, security threats [13] and regulatory pressures have pushed supply chain managers to adopt and adapt solutions that enable them to have a better understanding of their performance in a larger economy, environment and society [11]. They believed that visibility of the process and agents of the supply chain, transparency of steps and resources used, traceability of goods and verifying the authenticity of products play a significant role in the process of decision making [11].

Different tools, such as IoT and blockchain, may be used for making a smart supply chain, and little research has been conducted on this subject. There are still research gaps in this area of study, such as the study of relationship of these three areas: smart supply chain, IoT and Block chain [4]. Another application is the lack of categorization of blockchain and IoT features that affect making a smart supply chain. Motivated by the gap, this research addresses the following research questions: What key factors and contextual conditions influence the smart supply chain? What are the key features of IoT influencing the smart supply chain? What are the key features of blockchain influencing the smart supply chain? How can blockchain and IoT influence the smart supply chain patterns?

Therefore, finding criteria, components and features related to this matter will help sufficiently to reach a better understanding of such relationships. The features in a smart supply chain include the use of instruments, components interconnectedness, optimal decision making, automatedness, integrity of relationship and innovativeness. The features in IoT include data sharing, real-time analysis, traceability, identification and identity verification. Blockchain features also include decentralization, changeableness, traceability, and security and transaction facility [14].

In terms of contributions to the research, this paper presents the effect of IoT and blockchain on making a smart supply chain and assesses the effects and relationships of each factor with others and finds a pattern for it. This study aims at studying the application of IoT and blockchain for designing a smart supply chain and its role will also be investigated in the defense supply chain. The direct users of this study are the organisations where attention to safety issues, transparency and authenticity of products in their supply chain activities and traceability of such activities are in their agenda, and where the use of new technologies in their supply chain, such as IoT, is sought.

Both qualitative and quantitative methods were employed in this study. The qualitative method was Delphi technique [15,16], which was employed to choose indigenous components and proper features for the pattern; the quantitative method was Dematel [17], which was used to assess the relationships between the existing concepts in the pattern and access network structure between components. Delphi technique is a group knowledge acquisition method, which is also used for qualitative issue decision making. Delphi technique can be used for qualitative research that is exploratory and identifying the nature and fundamental elements of a phenomenon, which is the basis for study. Structure modeling method was also employed for the purposes of classification of the network structure obtained from Dematel technique.

The findings of this study indicate that optimal decision making and innovation are the most passive components in the formation of smart supply chain and are influenced more than other components in the pattern. Therefore, it is necessary that other components be realized at first so as to achieve optimal decision making, which is one of the most important goals of creating a smart supply chain. Considering the obtained results from the current study and the provided roadmap, it is first required to employ IoT and blockchain, and then to use instruments, i.e., to benefit from the data obtained in the previous step in order to implement the next step, which is connecting the components and integrating the relationships and processes. All the above-mentioned steps eventually lead to innovation in a smart supply chain and optimal decision making for an optimal performance.

The remainder of this paper is structured as follows. The next section presents theoretical foundations, followed by an introduction to the concept of smart supply chain, blockchain, internet of things and their relationships. The research method and details of our approach to data collection and analysis are then presented. Next, we present the research findings in the form of a framework. The paper concludes with a comparison of the findings with the literature, presents a summary of the contributions and discusses future research directions.

## 2. Theoretical Foundations

Supply chain services often play a key role in a firm’s ability to deliver customer value [18]. Among the key goals of an effective supply chain management involves getting the product in the right condition, in a timely manner and at the lowest possible costs [19]. Measurement of supply chain management performance is often described in terms of objectives such as quality, speed, dependability, cost and flexibility [18,19].

Due to increased competition, globalization and outsourcing, the number of players in a typical supply chain has increased significantly. In response, firms have introduced supplier evaluation programs using environmental and social criteria [20] as well as previous research arguing the need to carry out research on smart supply chains via taking advantages of new IoT and blockchain tool features [7,21].

Global supply chains are complex and face multiple uncertainties [21]. Despite the increasing use of IoT in supply chains, we still face many challenges in this regard. Most IoT challenges are related to security concerns and privacy [13]. Apart from this, there are other challenges: communication skills, lack of standards, legal challenges, regulatory issues, emerging economic issues and issues regarding system development [22]. It has also created fundamental blockchain technology, which takes the need for trust out of the equation. Prior to this, the demand for trust in large transactions was considered as a crucial challenge that has diminished with the advent of blockchain technology [7].

### 2.1. Smart Supply Chain

Smart supply chain management uses a large amount of data for better decision making, uses advanced technology and achieves a comprehensive insight during the operation. In other words, for smart supply chain management, all parts in the storeroom, distribution centres, stores and E-commerce portal should be connected and interact with each other. All mentioned parts should have the ability to exchange data and re-orders, if required, so that they will be informed of the customers’ needs at any place and time.

Several factors are critical to the smart supply chain. These include interoperability, visibility, transparency and stability of the supply chain, as well as energy efficiency; the practical strategy for making smart supply chain is to collect the relevant data and to do a quick analysis of the data for a better and deeper understanding of the current performance.

Furthermore, smart supply chain management may decrease the costs, increase the profitability and result in the success of companies. Nowadays, supply chain is operating in a complex and competitive environment, under the influence of the forces constantly changing the market and technology. Several issues are worth noting here, including different participatory entities, collective information and complex network structures. Supply chain users must specify how information technologies and new processes may help them conduct better activities.

In addition to minimizing their use of resources, the effective management of trading process in their transactions must be taken into consideration.

Using international standards and advanced technology, the smart supply chain changes the traditional system and process into a smart system. Interoperability, visibility and stability are the fundamental objectives in a smart supply chain. While the share of trade and logistics is more than 70% in a national economy, realizing an efficient supply chain management will help to better control and monitor physical shipments and information flow in a supply chain.

Wu et al. [4] believes that the supply chains will become more complicated, costly, uncertain and vulnerable. In order to effectively deal with the growing challenges, the supply chains have to be smarter. They pointed out that the new supply chain version proposes large-scale smart infrastructures for integrating data, information, physical objects, products and commerce. Hessman [23] noticed that the factories equipped with smart instruments could carry out tasks with global teams, dynamic systems and intelligent analytics all across the farthest steps of the value chain. Certainly, companies benefitting from the above-mentioned capabilities are facing the rivals who cannot make a profit. Unsurprisingly, there are many instances of application of smart supply chain, for example, smart transportation management system and smart factory.

A smart environment is defined as “a physical world that is richly and invisibly interwoven with sensors, actuators, displays and computational elements, embedded seamlessly in the everyday objects of our lives, and connected through a continuous network” [24]. IBM proposes especially three features (instrumentation, interconnectedness and smart) for the next generation smart supply chains.

The smart supply chain includes many characteristics, such as the internet of things, smart machines and smart infrastructure, interconnectedness, complete data collection and real-time communication across all stages of the supply chain, smart decision making, and efficient and responsive processes for offering better services to customers.

Wu et al. [4] pointed out that currently smart supply chain programs are quickly being developed and used. In essence, smart supply chain programs significantly offer many advantages that would not otherwise be available. For example, a number of data can be collected and applied to make better business decisions and increase performance and faster response. Furthermore, dynamic complexity has surpassed the possibility of human intervention to identify and solve many system problems. The smart supply chains can possibly eliminate many persistent insufficiencies. It is a challenging task to obtain higher efficiency and performance improvements through conventional perspectives, and organisations increasingly tend to develop newer technology-driven solutions and innovations based on business models. Smart devices (as new information technologies) can now support monitoring instrumentation, carrying out analysis and decrease costa remarkably [5].

Based on our explanations in terms of smart supply chain, we defined smart supply chains collectively with six distinct features: Instrumented, Interconnected, Optimal decision making, Automated, Integrated, and Innovative. Among all key resources, information systems still play a vital role in supply chain management because the supply chain performance is merely determined and facilitated by real-time collaboration. Complex integration of the supply chain management is impossible even without making progress in information and technology systems. According to Zhu et al. [5], smart supply chains exist to create value for their stakeholders and organisations through realising benefits at an optimal resource cost while optimising risk. More information, better decision making, better process and even better products are what a smart supply chain can and must produce.

### 2.2. Blockchain

Blockchain is a type of information and reporting system, a unique e-book and records what is worth being recorded in it. Its difference with other systems is that the information stored on this system is shared between network members, and by the use of encryption, is almost impossible to delete or manipulate the recorded information [25].

Blockchain technology offers many benefits, such as trust, independence, speed, stability, precision and effectiveness [26,27]. The unique features of a blockchain program (decentralization, autonomous, peer-to-peer relationship, unchangeable records and approval schedule) result in an increased productivity, time saving and money, data security assurance, transferer’s error elimination and the use of information. Decentralization means the absence of intermediaries or central authority; individual participants or the selected participants in one blockchain are able to verify the records of their trading partners and access the entire database and its full history, directly and without the help of intermediaries.

Additionally, blockchain eliminates the requirements of centralized management, by eliminating the role of the management intermediary. Peer-to-peer systems are distributed systems, made up of independent nodes (personal computers), which provide their computing resources (processing power, storage capacity or information distribution) directly to others. Indeed, in distributed system application, the blockchain technology has been designed to mitigate any single point of failure; customers do not have to trust their service providers [28]. When users join the peer-to-peer system, they turn their computers into system nodes, all of which are of equal importance. Although users may vary depending on the resources they use, all nodes in the system have same functionality. Therefore, the computers of all users are both suppliers and consumers of resources. As blockchain technology is a decentralized network, each participant or node is shared throughout the blockchain existence and has the same information or trading history. Blockchain maintains an unalterable track of records of trading on the leader system, making it impossible to forge after this event, because the information is not stored in one place, and is encrypted and shared across the network. With timed data, blockchain records the order of transactions and tracks data. The blockchain time sequence data feature can be used to track transaction information; this feature which can be widely used on the internet of things, is very inexpensive [29].

Blockchain uses an asymmetric encryption system to encrypt data. This system is divided into encryption and decryption keys, which can be separately stored and used without the use of secure channels. Blockchain guarantees the privacy of data by encrypting IoT data, which will result in more secure users’ privacy. Blockchain data has transparent, uncorrected, and unanalyzable features, and the use of blockchain technology in the process of product tracking can create a chain with clear tracking and sharing capabilities.

In this chain of transparent tracking and sharing, manufacturing companies, sellers, retailers or regulators cannot interfere or manipulate the relevant links. This ensures the accuracy of the tracked data and the reliability of IoT data. Blockchain technology adopts a distributed, decentralized approach and uses the asymmetric type of encryption to encrypt data, and the approval and consensus mechanism of blockchain can help prevent illegal or malicious internet nodes, which can effectively withstand the privacy of IoT. The IoT information exchange model is inefficient and has little effect if data is distributed. Current IoT models are heavily focused on developing vertical solutions, limited by hardware/software platforms and support [28]. Hence, IoT needs an open platform to exchange data in a secure environment. One-way information integrates complete and regular information in a library, which helps to understand the real-time situation from all aspects and contributes to the development of IoT. Blockchain’s decentralized architecture will change the IoT before the emergence of ongoing deadlock [30,31].

The blockchain network is scalable in nature because it is maintained by a network of counterparts. The more affiliates (or miners) that join the network, the greater the network’s computing capability would be [32]. Everything on the blockchain can be verified and validated, leading to more trust in the system, in other words, authentication [30,33].

### 2.3. Internet of Things (IoT)

In general, IoT refers to many things, including peripheral objects connected to the internet, and can be managed by apps on smartphones and tablets [4]. Most of IoT challenges are related to security and privacy concerns [33], such as activation sensors, raw data, the storage of processed data, and analytical and computational engines.

By transmitting radio signals from product identification, and other information, RFID technology may provide a comprehensive perspective of the supply chain [33], increases performance and improves the efficiency [34], to reduce the negative effects of organisational objectives [35]. Additionally Visich et al. [36] pointed out that the most important effects of the use of RFID are the effects of automation on operational processes, followed by information effects on management processes [36]. IoT smart devices can help supply chain companies to reduce the cost of obtaining information [37]. Because the companies have an option to acquire additional information about the demand by hiring experts, a commission fee must be paid to each hired expert, and there exists a trade-off between the cost and the value of the information [37].

The first important example of the use of industrial IoT, is logistic and supply chain management [38]. RFIDs can be attached to objects to identify materials and goods, such as clothing, furniture, equipment, food and liquids. Using RFIDs will help in efficient management by providing accurate knowledge. Moreover, the entire life cycle of objects can be traced.

In addition, real-time analysis by sensors allows the detection of product decay events, the sensors may monitor continuous temperature and humidity inside warehouses or cold storage, and the stimuli may correct them. Moreover, by using RF ID-based authentication processes, product integrity may be guaranteed. Other interesting programs on IoT are smart shopping systems. By tracking cell phones, these systems track users’ shopping habits and guide them in stores, supermarkets and discount centres or help with quick payment operations (for example, automatic checks using biometrics) [10]. Undoubtedly, there are concerns, such as low computing power and the storage capability of IoT devices, which may limit the use of blockchain. In addition, the authors assign a unique identity to each device through a blockchain-based IoT software. In this regard, Huh et al. [39] suggest using the Atrium platform to perform empowerment management within IoT. Blockchain can be a solution to the problems of the internet of things, using it to track billions of connected devices, process commands and coordinate devices. This decentralized approach eliminates the possibility of network failure and creates a more robust ecosystem for devices, as well as removing the concept of the IoT central server. The blockchain uses an encryption algorithm that keeps customer data confidential [40].

Managing an IoT device includes controlling configuration settings and performance modes, as well as ensuring uninterrupted performance. Controlling configuration settings and blockchain-based operating modes can prevent unauthorized access as well as protect against denial-of-service (DoS) attacks.

### 2.4. The Relationship between IoT, Blockchain and Supply Chain

Blockchain research has argued how we can accurately track and mange billions of connected devices, how to store big data generated by these devices, and how to do all of this safely and securely. Kamran et al. [41] reported that the decentralized approach eliminates the possibility of network failure and creates a more resilient ecosystem for devices. In addition, the encryption algorithm used in blockchain keeps customer data confidential. They believed that the acceptance of IoT consumers, and blockchain technology could be an answer to these challenges, in a way that is used to track billions of connected devices, process commands and enable coordination between devices.

Fernández-Caramés and Fraga-Lamas [42] pointed out that the relationship between blockchain and IoT is likely to be influenced by several elements. The first element, decentralization, examines blockchain plays a vital role to address privacy and security issues [12]. The second element, peer-to-peer system, examines blockchain as a comprehensive solution, peer-to-peer communication is often used for applications, such as mist computing and intelligent swarms. The third element focuses on the payment process, blockchain becomes popular for payment systems such as Bitcoin that eliminate the requirement to trust any third party, such as a bank. The last element, traceability, examines how blockchain will be helpful for auditing purposes. Combining blockchain and IoT is referred to as BIoT [42]. Therefore, blockchain technology has benefits for large-scale IoT systems, including proven and powerful data, reliability, recording of historical events, recording of old transactions in smart devices, the self-guidance of the performance license, the sharing of the distributed file, the destruction of the central control power of the unit, the reduction of costs in the development of large infrastructures of internet and the acceleration of transactions [43]. Further, blockchain-based solutions can assure the customer of the originality and high quality of the products and make them more willing to buy from the winner. Blockchain technology improves transparency and traceability in the supply chain through the use of immutable data, distributed reserves and controlled access to users [43].

Blockchain collaboration enhances real-time independent and secure payment services, enhances traditional commerce, e-commerce or public and private transportation systems. There are multiple examples of programs. In the future, IoT can be directly linked to a bank account based on cryptocurrencies to make micro-transactions in exchange for services, while similar approaches may be applied to the smart grid.

## 3. Research Methodology

**Figure 1:**
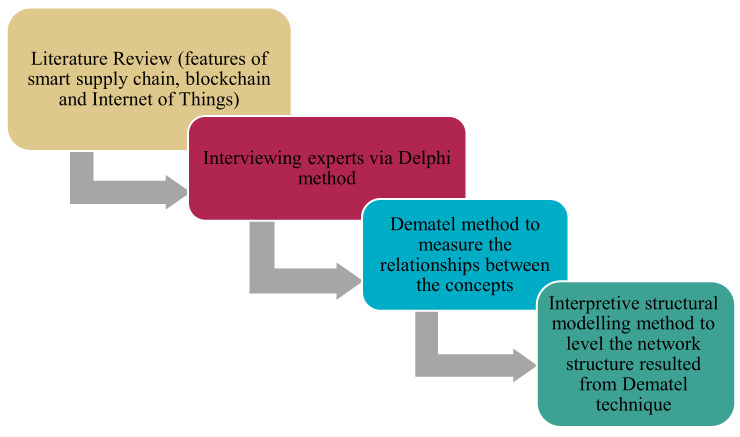
Research Method Structure.

In this research, the key features of smart supply chain, blockchain and IoT have been collected by a library method as influential and influential components in the smart supply chain model and by interviewing experts through the Delphi method [15,16]; the final components required for the pattern have been specified. Then, a quantitative method is used, which includes the Dematel method to measure the relationships between the concepts in the model and achieve the network structure between the components, and then the interpretive structural modelling method to level the network structure resulted from the Dematel technique, is outlined. The purpose of using the Dematel method is to find out the intensity of relationships between the components and their network structure, while the aim of using the interpretive structural modelling method is to level the hierarchical structure resulting from the Dematel technique and to achieve the access matrix and hierarchical structure as shown in Figure 1.

Delphi technique is used to “identify” and “screen” the most important decision-making indicators [15,16,44]. Although the Delphi technique is not a multi-criteria decision-making method, in many cases it is used to screen indicators or reach a compromise on the importance of decision-making indicators before using multi-criteria, decision-making techniques. Using the knowledge and expertise of a team to make decisions about issues of a qualitative nature is very helpful. Delphi technique is one of the methods to acquire group knowledge that is also used in decision making on qualitative issues. The Dematel technique is a decision-making technique based on multiple criteria and is a comprehensive method for analysing and creating a structured pattern based on cause-and-effect relationships in problems with a high number of components [17,45].

Linstone and Turoff [46] defined the Delphi technique as “a method for structuring a group communication process so that the process is effective in allowing a group of individuals, as a whole, to deal with a complex problem” (p. 3). The main purpose of the Delphi method is “to acquire the most reliable consensus of a group of expert opinions by a series of intensive questionnaires combined with controlled opinion feedback” (p. 458) [47]. By obtaining the consensus of a group of experts using the process, researchers can identify and prioritize issues and develop a framework to recognize them.

Habibi et al. [16] argued that there are various reasons for the use of the Delphi method, such as the requirement for experts’ judgement, general agreement to achieve the expected results, anonymity in data collection, a complex, multidimensional, and interdisciplinary problem, lack of consensus and imperfect knowledge, experienced and capable experts, dispersion of experts, no time limitation and lack of cost-effective method. This study uses a Delphi method, aligned with a qualitative research approach and helped interpret field data from the selected documents.

The Dematel technique transforms cause-and-effect relationships between components or indicators using graph theory into an understandable structural model [17]. This method is one of the best ones to measure communication, designing and building a strategic map, which is based on experts’ judgement. In order to implement this method, the following steps must be taken:

- Step 1—Selection of experts in the field of subject or problem
- Step 2—Identification of the criteria and components to be studied and the linguistic scale associated with them
- Step 3—Calculation of experts’ judgement

In order to measure the relationships between the components that make up the set of components C = {c*_i_*_|*i* = 1, 2, …, *n*_} the selected group of experts, including *k* = 1, 2, 3, …, *p*, in which “P” represents the members of the experts in step 1, is requested to compile a set of pairwise comparisons according to defined linguistic terms.

- Step 4—Initiation of direct-relation matrix

In this step, we form a direct-contact matrix using the initial Dematel matrix. This is the first step of the Dematel matrix, which is obtained from the Dematel questionnaire as shown in Appendix B. If there are several respondents, comments can be integrated using the arithmetic mean method. In this step, the Dematel decision matrix would be completed by using the spectrum Table 1.

- Step 5—Calculation of normal matrix

In this step, the direct-contact matrix of step 1 would be normalized. To normalize, the decision matrix values are divided by the largest total number of rows or columns.

- Step 6—Estimation of the total-relation matrix

First, the normal matrix is subtracted from the unit matrix (I), it then gets reversed and multiplied in the normal matrix to establish the total-relation matrix

- Step 7—Display of network-relation map

The last step in the Dematel method is to remove minor connections using the threshold value snd gain a network with high communications. Of course, in this step, the influence of factors can be calculated using receiver (R) and dispatcher (D) factors.

- Step 8—Draw the cause-and-effect diagram or Cartesian coordinates between the components, the seventh step is utilised.

The horizontal side of the diagram shows D + R, while the vertical side shows D−R. D represents the order of the elements that strongly influence other elements, and R indicates the order of the elements that are affected. Therefore, the order of the elements in the row indicates the range of the penetrating elements, and the order of the elements of the column represents the range of the elements to be penetrated.

The actual location of each element in the final sequence is determined by D + R and D−R columns, so that D−R indicates the position of an element on the axis of widths, and if it is positive, that factor is definitely an infiltrator, and if it is negative, that factor will definitely be a recipient or (under influence). D−R Indicates the total intensity of an element (along the longitudinal axis) both in terms of penetration and in terms of being affected. In other words, D + R is the extent to which the desired factor affects the model or system, and the larger it is, the more interaction with other system factors and the greater importance it has. D−R indicates the impact power of each factor. In general, if it is a positive number, the variable is considered cause and if it is negative, the variable is considered effect.

In the Cartesian coordinate system, the position of each component is determined by a point to the coordinates (D−R, D + R), and in this way, a graphic diagram of cause-effect relationships of the component is drawn. The interpretive structural modelling method was also utilised to level the network structure resulting from Dematel technique. In the following, the steps and algorithm of this method are fully described.

The researchers used both expert opinions and average opinions to achieve a direct relationship matrix of questionnaires. In order to avoid repetition to achieve the direct communication matrix in the structural interpretive modelling technique, the average comments have been used in this section. To do this, one cut was considered according to the threshold value of the desired cut equal to K = 0.125, and for all values greater than or equal to this cut, the matrix values of the perfect relations were obtained equal to one, and for all values less, considered as zero, and therefore, the direct-relation matrix is obtained from the total-relation matrix (direct and indirect communications). Due to the use of the results of the Dematel technique, the implementation of the interpretive structural modelling technique follows.

In this step, the total-relation matrix is separated. In this research, to separate the matrix levels from the division into possible levels, a structure has been used. Levels of a possible structure of an information system are considered as L_0_, L_1_, L_2, …_ L_k_ with L_0_ = 0. Then to separate these levels, there is the following equation:

$$L_{j}=\left\{N_{i}\in \left[N-L_{0}-L_{1}-L_{2}-\ldots -L_{j-1}\right]/R_{j}\left(N_{i}\right)=R_{j}\left(N_{i}\right)\cap A_{j}\left(N_{i}\right)\right\}$$

In last step, the diagram obtained from the results of the previous step divisions is drawn.

$$\left\{\begin{array}{l} R_{j}\left(N_{i}\right):\\ A_{j}\left(N_{i}\right): \end{array}\right.$$

All vertices whose columns in T have input 1 in the row i.

All vertices whose rows in T have input 1 in the row I = j.

According to the explanations given above, the literature review, and interviews with experts in the Delphi method, we came up with a framework for smartening the supply chain, which by interviewing experts, localizes the characteristics and effective components of blockchain and IoT on the smart supply chain (see Appendix A).

Based on the results obtained from the research, and the opinion of experts, the components can be presented as follows:

- Smart supply chain features (instrumented, interconnected, automated, integrated, innovative)
- Blockchain features (decentralized, unchangeable, traceable, secure, transactions facilitator)
- IoT features (sharing of information, real-time analysis, tracking and identification, authentication)

### 3.1. Smart Supply Chain Features

- Instrumented: The next generation supply chain increasingly focuses on sensors, RFID and meters concepts.
- Interconnected: Smart supply chain is interconnected in some concepts, such as businesses and assets, IT systems, products and other smart objects.
- Optimal decision making: Smart supply chain makes major optimal decisions for increasing organisational performance.
- Automated: Smart supply chains should automatically replace other low-efficiency resources, such as the workforce with machinery in much of their process flow.
- Integrated: Supply chain process integration facilitates communication and collaboration in all stages, making important decisions, and sharing information and knowledge in all stages.
- Innovative: Innovation in supply chain concept means coming up with new ways to do things. It can develop new values through solutions that meet new requirements and create more effective products and ideas.

### 3.2. Blockchain Features

- Decentralization: it means the absence of intermediaries or central authority; each of the selected participants or participants in a blockchain has the ability to verify the records of their trading partners and access the entire database and its full history directly and without the help of intermediaries. Essentially, blockchain eliminates the need for centralized management requirements by removing the need for a mediating role for trust.
- Unchangeable: Because blockchain technology is a decentralized network, each participant or node is shared throughout the blockchain’s existence and has the same information or trading history. Blockchain retains an unchanging track record of trading on the leader system, and this makes it impossible to forge after this event because the information is not stored in one place and is encrypted and distributed to everyone on the network.
- Traceable: Blockchain data has clear, uncorrected, and unanalysable features, and the use of blockchain technology in the product tracking process can create a chain with clear tracking and sharing capabilities. In this chain of transparent tracking and sharing, manufacturing companies, sellers, retailers or regulators cannot interfere or manipulate the relevant links. It also helps to understand the product originality, in addition, the time and place of these actions can be determined.
- Secure: Blockchain technology adopts a distributed, decentralized approach and uses the asymmetric type of encryption to encrypt data. The blockchain technology approval and consensus mechanism can help prevent illegal or malicious internet nodes, which can effectively provide the privacy of IoT.
- Transactions Facilitator: Blockchain collaboration enhances real-time independent and secure payment services, enhances traditional commerce, e-commerce or public and private transportation systems. Safe transfer of digitally signed documents can verify the assets and identities of individuals and minimize the need for physical interactions and communications. In the future, IoT devices can be directly linked to a cryptocurrency-based bank account so that micro-exchanges can be made in exchange for services. Combining smart contracts with blockchain increases reliability, security and flexibility.

### 3.3. IoT Features

*Tracking and Identification:* with IoT, radio frequency identification tags, sensors, barcodes, labels and GPS chips, products, packages and containers for shipping, warehousing, customer sales and disposal can be tracked and located at any time. *Real-time Analysis:* while sensors help for identification, stimuli can help improve performance. For example, real-time analysis by sensors allows the detection of product decay events, which are vital for food and liquids. *Information Sharing:* based on broad communication, IoT provides easy access to extensive information resources, which supports more comprehensive intelligent services. *Authentication:* through an IoT software, each device is assigned a unique identity so that it can retrieve data and work on it.

Before starting the Dematel algorithm, for ease of operation, the components were named according to the Table 2.

The Dematel questionnaire was completed by 12 experts as shown in Table 3, including managers and experts in the field of supply chain, internet of things and blockchain. Face validity has been used for this research. To validate the reliability of this research using SPSS software and based on Cronbach’s alpha method, the validity of the questionnaire was measured.

**Figure 2:**
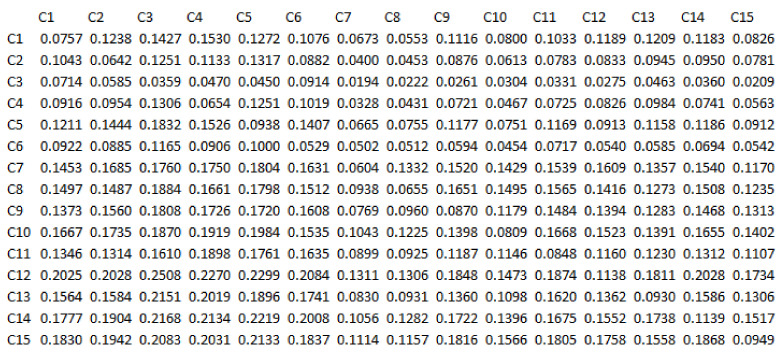
Complete Relationship Matrix Diagram.

According to the fourth step of the Dematel algorithm, the mean matrix is calculated and then the mean of the average direct relation among the components is normalized based on the fifth step of the Dematel algorithm. It is important to note that in the questionnaire, direct relationships between the components were questioned, so in order to achieve indirect relationships, the sixth step of the Dematel algorithm has been used. All related calculations are shown in Table 4, Table 5, Table 6 and Table 7 while the intensity of the relationship between components is based on the matrix in Figure 2.

The sum of rows and columns is calculated for every single row and column of this matrix. Dispatcher (D) indicates the order of elements that strongly influence other elements, while receiver (R) indicates the order of the elements that are affected. So the order of the elements in the row indicates the order of the penetrating elements, and the order of the elements of the column will indicate the order of the affected elements. Calculations are provided in the following tables from number 4 to 7.

From the provided information, it turns out that C_12_ (sharing of information) is the most penetrating component in the pattern, followed by C15 and C14 respectively. On the other hand, C3 (optimal decision-making), is the most affected component in the system. From the eighth step, the sum and difference of rows and columns of the total-relation matrix is calculated. The sum of the rows and columns is equal to D + R, while the difference is D−R.

D + R indicates the extent to which the desired factor affects or get affected in the pattern or system, and the larger it is, the more interaction with other factors and the greater importance it has, while D−R indicates the impact power of each factor. In general, if D−R is a positive number, the variable is cause and if it is negative, the variable is effect. The values of D + R and D−R are as follows.

**Figure 3:**
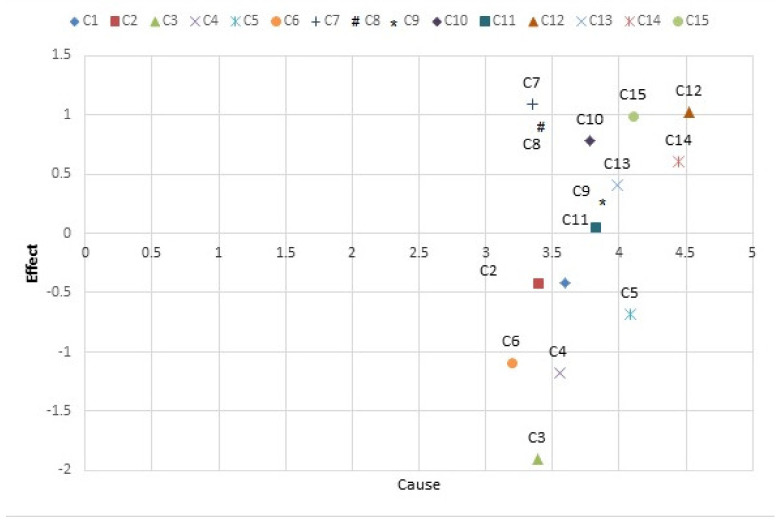
The relationship between cause and effect.

In Descartes’ coordinates, the position of each component (C), with a set of coordinates (D + R, D−R) is specified. In this way, a graphic diagram of the cause–effect relationship of the components is made. Figure 3 shows the graphic diagram of the components.

In this figure, the row and column difference of total-relation matrix (D−R) shows the components C1, …, C6 are considered as effect components while C7, …, C15 are cause components and among components, C12 is the most common cause and C3 is the most common effect, respectively.

**Figure 4:**
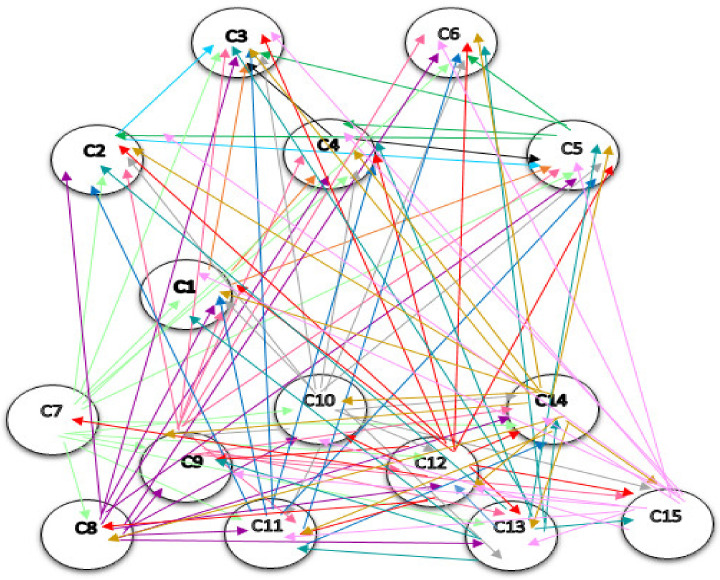
The network structure among the components. C1: Instructed. C2: Interconnected. C3: Optimal Decision Making. C4: Automated. C5: Integrated. C6: Innovative. C7: Decentralized. C8: Unchangeable. C9: Traceable. C10: Secure. C11: Transactions Facilitator. C12: Sharing of Information. C13: Real-time Analysis. C14: Tracking & Identification. C15: Authentication.

The network structure among the components of the pattern, resulting from Dematel, is shown in Figure 4. It should be noted that in the network structure, only direct relations that are the results obtained from the questionnaires, are drawn, and indirect relations in this structure are not shown. If they are intended to display, they should be displayed as dotted lines.

**Figure 5:**
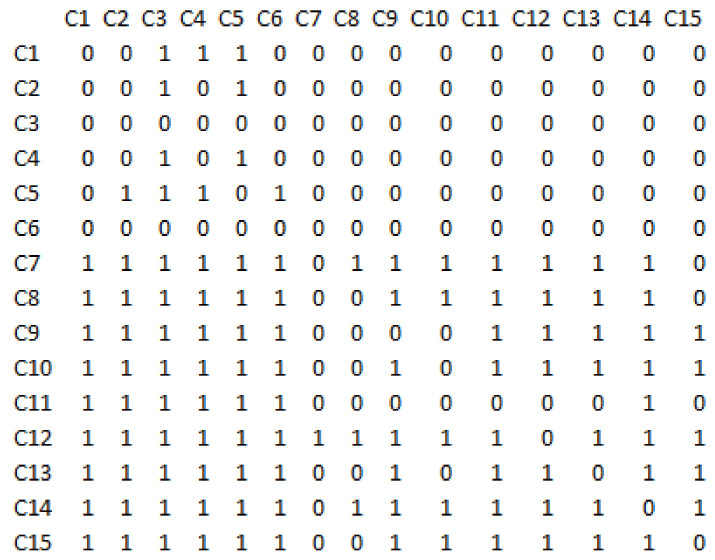
The direct-relations matrix in the structural interpretive modelling technique.

The interpretive structural modelling technique was applied in order to level the network structure resulting from Dematel. The direct-relations matrix is obtained from the total-relations matrix (direct and indirect communications), which is shown in Figure 5. To do this, a cut is used according to the threshold value, which was considered equal to K = 0.125, and for all values greater than or equal to this cut, the values of the obtained total-relations matrix are equal to one. And for less, considered as zero.

**Figure 6:**
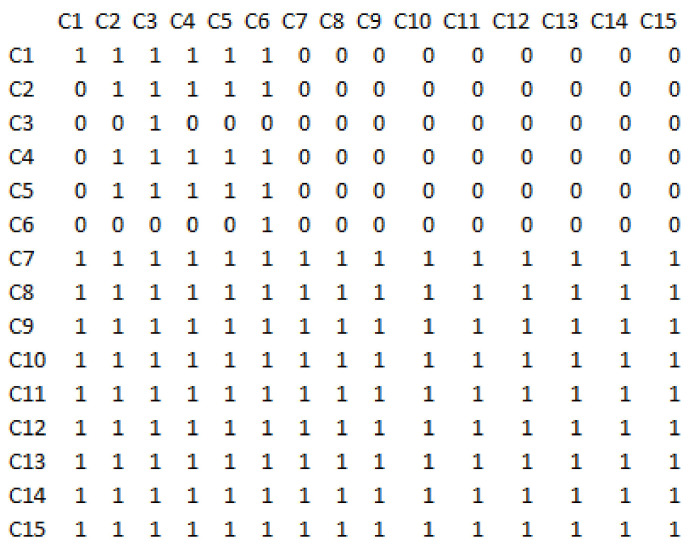
The total-relation matrix in the structural interpretive modelling technique.

Using Boolean algebra and the relations described, the total-relation matrix in the structural interpretive modelling technique is calculated and shown in Figure 6.

**Figure 7:**
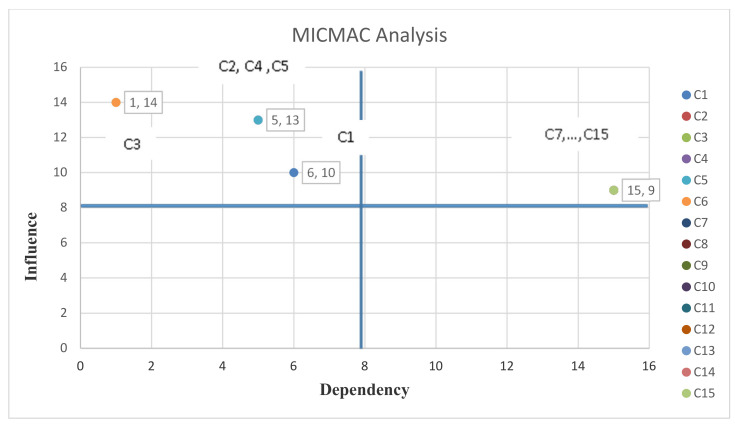
Interpretive structural modeling and MICMAC analysis.

Now, using the total-relation matrix, MICMAC analysis of interpretive structural modelling can be carried out. MICMAC analysis is one of the topics of structural-interpretive modelling. Based on the power of dependence and influence of variables, the coordinate system can be defined and divided into four equal parts. MICMAC analysis is based on the power of influence and the degree of dependence of each variable and allows further scrutiny of each variable as shown in Figure 7.

- Influence—The number of elements affected by the i-th element.
- Dependency—The number of elements that affect the i-th element.

In this analysis, the variables are divided into four groups: autonomous, dependent, interconnected and independent.

- Autonomous: Autonomous variables have low dependency and conductivity. They are generally separated from the system because they have poor connections to the system. A change in these variables does not cause a serious change in the system.
- Dependent: Dependent variables have strong dependencies and poor guidance. These variables are generally highly affected, while they have little impact on the system.
- Interconnected: These variables have a high dependency and high conductivity, in other words, they can highly impact and be highly affected, and any small change on these variables causes fundamental changes in the system.
- Independent: Independent variables have low dependence and high guidance. In other words, having high impact and getting low effect are the characteristics of these variables.

Therefore, the C1 to C6 components are in the dependent group, which means that they have strong dependence and poor guidance as shown in Figure 7. These variables are basically high and have little effect on the system, and the C7 to C15 components are in the interconnected group, which means they have high dependence and high guiding power. More precisely, every small change in these variables causes fundamental changes in the system.

In the following process, step six is used to separate the matrix *T_ISM_* and to determine the levels of the network structure resulting from the Dematel output. To separate the total-relation matrix, the two sets are defined as follows.

*Ri* includes all vertices whose columns in *T_ISM_* have an input equal to one in row I, and *Di* includes all vertices whose columns in *T_ISM_* have an input equal to one in row *i* = *j*. Then, the similarity of the two sets is calculated. If this similarity is equal to the set *Di*, then *i*-th component is levelled and after a complete repetition for all the components, it will be removed from the components in the next repetition. This process continues until all components are levelled. In the following table, *Ri* and *Di* are calculated for matrix *T_ISM_* (see Table 8).

**Figure 8:**
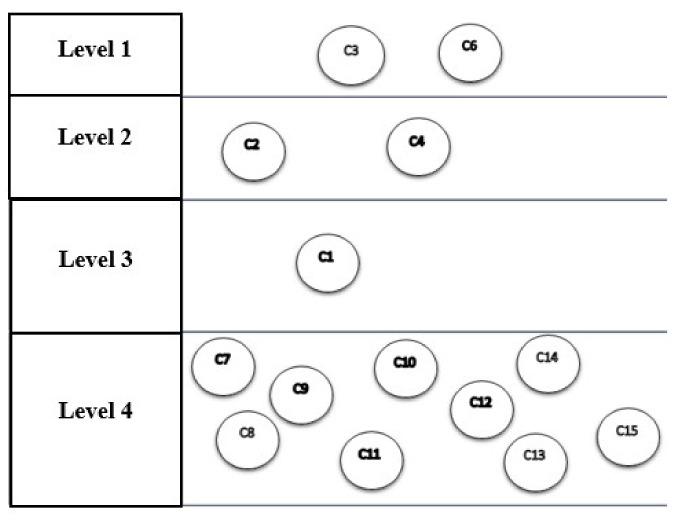
The network structure of the components and their levelling.

Table 9 shows that components C3 and C6 are at level 1. By removing these components, the second iteration is continued with the remaining components, and its output also shows the second level components (see Table 9). With the fourth iteration, the process ends. In the seventh step of the diagram, the results of the divisions of the previous steps are plotted, shown in Figure 8.

**Figure 9:**
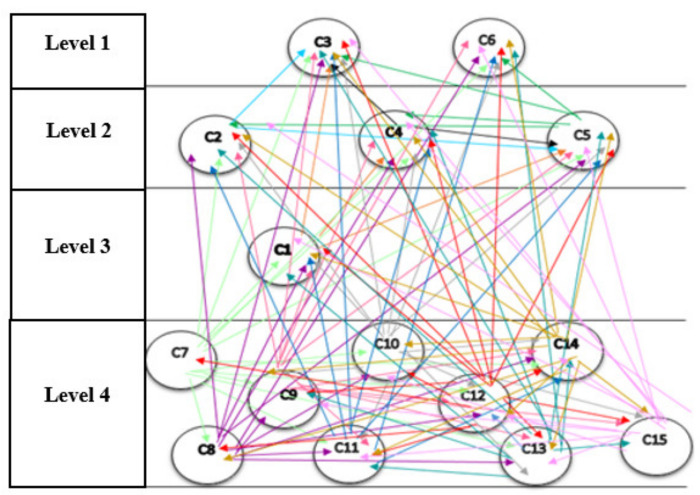
Output of Dematel technique and structural interpretive modelling. C1: Instructed. C2: Interconnected. C3: Optimal Decision-Making. C4: Automated. C5: Integrated. C6: Innovative. C7: Decentralized. C8: Unchangeable. C9: Traceable. C10: Secure. C11: Transactions Facilitator. C12: Sharing of Information. C13: Real-time Analysis. C14: Tracking & Identification. C15: Authentication.

It is important to take into account that the network structure of the components (output of Dematel technique) and their levelling (output of structural interpretive modelling) are all shown in Figure 8 and Figure 9. The levels that appear in the technique of interpretive structural modelling indicate the severity of the effect of the components, respectively. This means that the first level shows the most common effect level and the last level shows the most common cause level (see Figure 9).

## 4. Discussion

Dispatcher indicates the order of the elements that strongly influence other elements; therefore, the “information sharing” component has the most impact on other components. This is consistent with the empirical evidence in the research of Wu et al. [4], which noticed that knowledge sharing is a key driver of supply change performance.

The second component with high penetration intensity is the “authentication” component. This is consistent with the study of Bendavid and Cassivi [48], which pointed out that main impacts of applying RFID are automation impact on operational and management processes. Using smart devices (such as IoT) can help supply chain organisations to mitigate the cost of collecting data.

The third component that has the highest penetration intensity in the model is the “tracking and identification” component, complying with a study by Bendavid and Cassivi [48], which suggested that RFID technology provide a comprehensive perspective of the supply chain through RF signal transmission from product identification and other information. Indeed, RFID improves production efficiency and effectiveness as well as mitigates the negative theft and disaster effects.

On the other hand, receiver indicates the order of the elements that are affected; therefore, the “optimal decision making” component is most strongly influenced by the other components. This seems to be the main purpose of using a smart supply chain, smart supply chain management, to use a large amount of data to make better decisions, use advanced technology and gain full insight during the operation. Therefore, it helps managers to make the best decisions in difficult situations and to make optimal decisions on a large scale to optimize performance.

The second most strongly affected component is the “automation” component. According to Bendavid and Cassivi [48], automation is usually equated with two interconnected reasons: control costs and optimize processes. It is argued that the automation element has the potential to produce greater understanding of the supply chain phenomena; by connecting devices to the IT system through software, information is generated and insight is achieved. These devices are considered as part of a coherent intelligent network that can be utilised to automate the architecture of important information and even physical products. These schemes exist to create value for their stakeholders and organisations. Value creation means realising benefits at an optimal resource cost while optimising risk. Benefits can take many forms, e.g., financial for commercial enterprises or public service for government entities, so organisations have been integrating the devices with wider sensors and networks for years. Consequently, research has argued that much can still be learnt from studies of smart supply chain and usage [36,48].

The third component that has been influenced by other components is the “integration” component. Visich et al. [36] pointed out that companies are increasingly required for proper system integration to manage large data sets and then turn them into real-time smart decisions to increase performance.

Row and column differences indicate to what extent a factor gets affected; if this difference is a positive number, the variable is a cause variable, and if it is negative, the variable is considered an effect variable.

The six components of “optimal decision making”, “innovation”, “integration”, “interconnection”, “automation” and “instructed” were classified in the group of effect components, which are the goals of implementing the smart supply chain, and are at high levels. It should be noted that in fact, these are the components and features and capabilities of the smart supply chain that we need to achieve. Therefore, the causes produce the information we need and affect the use of tools, which is the use and application of that information, and then the instruction leads to the integration, automation and innovation of components, which are the main factors in implementing innovation and optimal decision making in the proposed model.

“Decentralization”, “information sharing”, “authentication”, “immutability”, “security“, “tracking and identification”, “real-time analysis”, “traceability” and “facilitation in transactions” are in the category of cause components and at low levels. Therefore, in order to implement a smart supply chain, it first needs to implement these components, which are in fact the same features and components of the internet of things and blockchain. So, by integrating IoT tools and using the blockchain platform, a big step can be taken towards smartening supply chains.

The interpretive structural modelling technique has divided the components into four levels according to the severity of the cause-and-effect relationship, the first level of which includes optimal decision making and innovation, The second level includes component interconnection, automation and integration; the third level involves the use of tools; and the fourth level also includes decentralization, information sharing, authentication, immutability, security, tracking and identification, real-time analysis, traceability and facilitation of transactions, all of the components in the fourth level are IoT and blockchain features (see Table 8).

## 5. Conclusions and Recommendation

Despite statements from previous sources and given the importance of the supply chain, its intelligence, concerns of firms in finding optimal large-scale decisions, increasing competition among firms and its significance as the most important source of competitive advantage in organisations and countries, research on supply chain intelligence with the internet of things in the blockchain platform is still limited with few studies reporting the key success factors for smart supply chain implementation. Systematically, the subject of smart supply chain and the effects of the IoT and blockchain on it has not been studied and no specific model has been provided for it. In this study, we attempted to provide a framework for smartening the supply chain with the IoT tool in the blockchain platform, which can be seen in Figure 9. The features obtained for the IoT and blockchain will be important and fundamental factors for the use of organisations and enterprises, and by using this framework, each company can use the IoT and blockchain features to make its supply chain smart according to its needs.

Future proposals for research include the impact of factors on each other, the use of system dynamics to model known factors, prioritization of each factor, reviewing the impact of these factors in a selected organisation and reviewing the implementation of the model as well as measuring the success rate of the model in achieving the defined mission for that organisation as a pilot.

## Figures and Tables

**Table 1:** The spectrum used for the method.

Equivalent	Effect
0	Non-effect (N)
1	Very Low (VL)
2	Low (L)
3	High (H)
4	Very High (VH)

**Table 2:** Variables coding.

Variables Coding			
C1: Instructed	C5: Integrated	C9: Traceable	C13: Real-time Analysis
C2: Interconnected	C6: Innovative	C10: Secure	C14: Tracking & Identification
C3: Optimal Decision Making	C7: Decentralized	C11: Transactions Facilitator	C15: Authentication
C4: Automated	C8: Unchangeable	C12: Sharing of Information	

**Table 3:** Participants in Demetel method and structural interpretive modeling.

Participants	Case Studies	Respondents
Academic people	Faculty	5
Industry people	Senior manager	4
	Project manager	3

**Table 4:** The sum of rows.

C1	C2	C3	C4	C5	C6	C7	C8	C9	C10	C11	C12	C13	C14	C15
1.588	1.29	0.611	1.188	1.705	1.055	2.218	2.157	2.051	2.282	1.938	2.774	2.198	2.529	2.545

**Table 5:** The sum of columns.

C1	C2	C3	C4	C5	C6	C7	C8	C9	C10	C11	C12	C13	C14	C15
2..009	2.099	2.518	2.363	2.384	2.142	1.133	1.27	1.812	1.498	1.883	1.749	1.792	1.922	1.557

**Table 6:** The sum of rows and columns (D + R).

C1	C2	C3	C4	C5	C6	C7	C8	C9	C10	C11	C12	C13	C14	C15
3.597	3.389	3.129	3.551	4.089	3.197	3.351	3.427	3.863	3.78	3.821	4.522	3.989	4.451	4.102

**Table 7:** The difference of rows and columns (D−R).

C1	C2	C3	C4	C5	C6	C7	C8	C9	C10	C11	C12	C13	C14	C15
−0.421	−0.809	−1.907	−1.174	−0.68	−1.087	1.089	0.888	0.24	0.784	0.054	1.025	0.406	0.607	0.988

**Table 8:** The four levels of interpretive structural modelling techniques.

Level	Name	Components
1	Realization of smart supply chain	Optimal decision making, innovation
2	Information technology and process automation	Interconnection, automation and integration
3	Use of information and technological tools	Instructed
4	IoT and Blockchain Components	Decentralization, information sharing, authentication, immutability, security, tracking and identification, real-time analysis, traceability and facilitation of transactions

**Table 9:** The first iteration, and its output indicates the components that are at level one.

*C_i_* Component	*R_i_*	*D_i_*	*R_i_* ∩ *D_i_*	Level Component
C1	C1, C7, …, C15	C1, …, C6	C1	-
C2	C1, C2, C4, C5, C7, …, C15	C1, …, C6	C2, C4, C5	-
C3	C1, C2, C3, C4, C5, C7, …, C15	C3	C3	C3
C4	C1, C2, C4, C5, C7, …, C15	C2, …, C6	C2, C4, C5	-
C5	C1, C2, C4, C5, C7, …, C15	C2, …, C6	C2, C4, C5	-
C6	C1, C2, C4, C5, C6, C7, …, C15	C6	C6	C6
C7	C7, …, C15	C1, …, C15	C7, …, C15	-
C8	C7, …, C15	C1, …, C15	C7, …, C15	-
C9	C7, …, C15	C1, …, C15	C7, …, C15	-
C10	C7, …, C15	C1, …, C15	C7, …, C15	-
C11	C7, …, C15	C1, …, C15	C7, …, C15	-
C12	C7, …, C15	C1, …, C15	C7, …, C15	-
C13	C7, …, C15	C1, …, C15	C7, …, C15	-
C14	C7, …, C15	C1, …, C15	C7, …, C15	-
C15	C7, …, C15	C1, …, C15	C7, …, C15	-

## Data Availability

Not applicable.

## Appendix A

**Table A1:** Summary of provision of model and data analysis.

Feature of Smart Supply Chain	Impact of Blockchain	Source	Impact of IoT	Source
Instructed	Information security	Subramanian [49]	Improvement of information accuracy with RFID	Bendavid and Cassivi [48]
	Security of wireless sensors with peer-to-peer, decentralized and secure connection	Daza et al. [28]	The technological revolution in computing	Atzori et al. [50]
	Confidentiality of information with encryption algorithm	Kumar and Mallick [40]	Product identification and unprecedented vision by carrying radio signals	Masciari [10]
			Identification, locating and tracking capability	Wu et al. [4]
			Measurement of key supply chain management processes	Kshetri [18]
Interconnection	Communication and messaging	Levine [51]	Real-time Cooperation	Wu et al. [4]
	The security of the IoT network with a peer-to-peer, decentralized and secure relationship	Daza et al. [28]	Network connection with the ability to exchange your information and environment	Wu et al. [4]
	Network malfunction elimination with decentralized feature	Daza et al. [28]	The technological revolution in communication at any time, place, media, and thing	Atzori et al. [50]
			Easy access to extensive database	Wu et al. [4]
Optimal DecisionMaking	Tracking of goods, ownership records and changing the status of assets for non-formal features of change	Christidis and Devetsikiotis [52]	Product identification and unprecedented vision by carrying radio signals	Masciari [10]
	Tracking of shipments and the process of transformation and progress of work for the unchangeability feature	Koetsier [53]	Ability to monitor and manage performance	Wu et al. [4]
	Ability to trace the source of raw materials and provide the basis for the final product or service with immutability and distribution	Francisco and Swanson [54]	Smart purchase	Borgia [38]
	Track billions of connected devices and process commands and coordinate between devices	Borgia [38]	Sensors real-time analysis	Borgia [38]
Automation	Help maintain processes on the blockchain, which is a prelude to simplification automation, and costs reduction	Casino et al. [6]	Acceleration with the help of sensors, robots and machines	Kshetri [18]
	Ability to create smart contracts by giving virtual identity to Traders and products	Francisco and Swanson [54]		
Integration	Automated device synchronization that leads to scalability	Casino et al. [6]	Real-time cooperation	Wu et al. [4]
	Automatic and decentralized methods cause high scalability	Li and Zhang [56]; Sharma et al. [57]; Sakakibara et al. [58]	Easy access to extensive database	Ma [55]
	Cooperation; Real-time independent and secure payments	Christidis and Devetsikiotis [52]	Authentication	Borgia [38]
	Intermediaries elimination and creation of an integrated structure	Subramanian [49]		
	Communication and messaging	Levine [51]		
	Transparency, information sharing, visibility andflexibility	Lamming et al. [59]		
	Direct communication between buyers and sellers without intermediaries	Subramanian [49]		
	Eliminate administrative and paperwork formalities by digital signature and authentication and minimize interactions	Kshetri [18]		
	Tracking and transparency with unchangeable data, distributed reserves and controlled access	Francisco and Swanson [54]		
Innovation	Authentication	Kshetri [18]	Authentication	Borgia [38]
	Unchangeable	Koetsier [53]; Francisco and Swanson [54]	Smart purchase	Borgia [38]
	Encrypted algorithm	Kumar and Mallick [40]	Radio signals	Masciari [10]
	Peer-to-peer relationship; decentralized and distributed	Daza et al. [28]	Sensors real-time analysis	Kshetri [18]; Borgia [38]
	Smart contract	Francisco and Swanson [54]		
	Virtual identity	Francisco and Swanson [54]		
	Digital signature	Kshetri [18]		

## Appendix B

Subject: Study of the Effects of the Relationships in the Smart Supply Chain Model

This questionnaire is designed to study the severity of effectiveness of the concepts in the smart supply chain model. The questionnaire aims to question the smart supply chain factors relying on the IoT tools in the blockchain platform, obtained based on the literature review. Please mark your opinion about every component as explained in the following. We thank you in advance for your time and attention.

**Name and Surname (Not Obligatory):** **Telephone No.:**				
				**Email Address:**
Project Manager ☐	Faculty member ☐	Counselor ☐	Senior Manager ☐	**Job title:**
over 15 years ☐	10–15 years ☐	5–10 years ☐	Fewer than 5 years ☐	**Work Records:**
over 40 years ☐	35–40 years ☐	30–35 years ☐	below 30 years ☐	**Age:**
Others ☐	University ☐	Research Institute ☐	Industry ☐	**Place of work:**
Others ☐	Blockchain ☐	Internet of Things ☐	Supply chains ☐	**Research areas:**

It is to be noted that the DEMATEL technique was employed to assess the severity of the interactive relationships between the components in the model. The DEMATEL technique is one of the decision-making methods, which is based on paired comparisons using the (0,1,2,3,4) spectrum for comparison, and by benefitting from expert opinions in the extraction of the factors of a system and systematic structuring, employing principles of graph theory, provides a hierarchical structure of the existing factors along with their cause-and-effect relationships. The severity of the mentioned relationships is expressed as numbers. Generally, it can be stated that the DEMATEL technique is used to identify and investigate the interrelationship between criteria and to construct a network relationship mapping.

Five Scale Spectrum of DEMATEL Technique and Definite Equivalent for Verbal Expressions.

**Very Significant Effect**	**Significant Effect**	**Small Effect**	**Very Small Effect**	**Ineffective**
**4**	**3**	**2**	**1**	**0**

**Ineffective (0)—Very Small Effect (1)—Small Effect (2)—Significant Effect (3)—Very Significant Effect (4)**			**Smart Supply Chain Characteristics**						**Blockchain Features**					**IoT Features**			
			**1**	**2**	**3**	**4**	**5**	**6**	**7**	**8**	**9**	**10**	**11**	**12**	**13**	**14**	**15**
			**Instrumented**	**Interconnectedness**	**Optimal Decision Making**	**Automated**	**Integrated**	**Innovative**	**Decentralized**	**Unchangeable**	**Traceable**	**Secure**	**Transaction facilitator**	**Sharing**	**Real-Time Analysis**	**Traceability & Identification**	**Identity Verification**
Smart Supply Chain Characteristics	1	Instrumented	0														
	2	Interconnectedness		0													
	3	Optimal decision making			0												
	4	Automated				0											
	5	Integrated					0										
	6	Innovative						0									
Blockchain features	7	Decentralized							0								
	8	Unchangeable								0							
	9	Traceable									0						
	10	Secure										0					
	11	Transaction facilitator											0				
IoT Features	12	Information sharing												0			
	13	Real-time analysis													0		
	14	Traceability and identification														0	
	15	Identity verification															0

## References

[1] Akhavan P., Elahi B., Jafari M. (2014). A new integrated knowledge model in supplier selection. Educ. Bus. Soc. Contemp. Middle East. Issues.

[2] Akhavan P., Namvar M. The mediating role of blockchain technology in improvement of knowledge sharing for supply chain management. Manag. Decis..

[3] Bidgoli H. (2010). The Handbook of Technology Management, Supply Chain Management, Marketing and Advertising, and Global Management (2).

[4] Wu L., Yue X., Jin A., Yen D.C. (2016). Smart supply chain management: A review and implications for future research. Int. J. Logist. Manag..

[5] Zhu X., Mukhopadhyay S.K., Kurata H. (2012). A review of RFID technology and its managerial applications in different industries. J. Eng. Technol. Manag..

[6] Casino F., Dasaklis T.K., Patsakis C. (2019). A systematic literature review of blockchain-based applications: Current status, classification and open issues. Telemat. Inform..

[7] Laurence T. (2019). Blockchain for Dummies.

[8] Zachariadis M., Hileman G., Scott S.V. (2019). Governance and control in distributed ledgers: Understanding the challenges facing blockchain technology in financial services. Inf. Organ..

[9] Cai H., Da Xu L., Xu B., Xie C., Qin S., Jiang L. (2014). IoT-based configurable information service platform for product lifecycle management. IEEE Trans. Ind. Inform..

[10] Masciari E. (2012). SMART: Stream monitoring enterprise activities by RFID tags. Inf. Sci..

[11] Huckle S., Bhattacharya R., White M., Beloff N. (2016). Internet of things, blockchain and shared economy applications. Procedia Comput. Sci..

[12] Hwang J., Choi M.I., Lee T., Jeon S., Kim S., Park S. (2017). Energy prosumer business model using blockchain system to ensure transparency and safety. Energy Procedia.

[13] Idrees S.M., Nowostawski M., Jameel R., Mourya A.K. (2021). Security Aspects of Blockchain Technology Intended for Industrial Applications. Electronics.

[14] Teslya N., Ryabchikov I. Blockchain-based platform architecture for industrial IoT. Proceedings of the 2017 21st Conference of Open Innovations Association (FRUCT).

[15] Ahmad M., Salah K. (2018). IoT security: Review, blockchain solutions, and open challenges. Future Gener. Comput. Syst..

[16] Habibi A., Sarafrazi A., Izadyar S. (2014). Delphi Technique Theoretical Framework in Quality active Research1. Int. J. Eng. Sci..

[17] Rana N.P., Dwivedi Y.K., Hughes D.L. Analysis of challenges for blockchain adoption within the Indian public sector: An interpretive structural modelling approach. Inf. Technol. People.

[18] Kshetri N. (2018). 1 Blockchain’s roles in meeting key supply chain management objectives. Int. J. Inf. Manag..

[19] Goldbach M., Seuring S., Back S. (2003). Coordinating sustainable cotton chains for the mass market–The case of the German mail order business OTT. Greener Manag. Int..

[20] Beske P., Koplin J., Seuring S. (2008). The use of environmental and social standards by German first-tier suppliers of the Volkswagen AG. Corp. Soc. Responsib. Environ. Manag..

[21] Koplin J., Seuring S., Mesterharm M. (2007). Incorporating sustainability into supply management in the automotive industry: The case of the Volkswagen AG. J. Clean. Prod..

[22] Rose K., Eldridge S., Chapin L. (2015). The internet of things: An overview. Internet Soc..

[23] Hessman T. (2013). The dawn of the smart factory. Ind. Week.

[24] Weiser M., Gold R., Brown J.S. (1999). The origins of ubiquitous computing research at PARC in the late 1980s. IBM Syst. J..

[25] Drescher D. (2017). Blockchain Basics, A Non-Technical Introduction in 25 Steps.

[26] Akhavan P., Philsoophian M., Rajabion L., Namvar M. Developing a Block-chained knowledge management model (BCKMM): Beyond traditional knowledge management. In Proceedings of the 19th European Conference on Knowledge Management (ECKM 2018).

[27] Morabito V. (2017). Business Innovation through Blockchain.

[28] Daza V., Di Pietro R., Klimek I., Signorini M. CONNECT: CONtextual NamE disCovery for blockchain-based services in the IoT. Proceedings of the 2017 IEEE International Conference on Communications (ICC).

[29] San K.M., Choy C.F., Fung W.P. (2019). The potentials and impacts of blockchain technology in construction industry: A literature review. IOP Conference Series: Materials Science and Engineering.

[30] Bambara J.J., Allen P.R., Iyer K., Madsen R., Lederer S., Wuehler M. (2018). Blockchain: A Practical Guide to Developing Business, Law, and Technology Solutions.

[31] Yang Y., Chen J., Liu M. (2018). Application of blockchain in internet of things. International Conference on Cloud Computing and Security.

[32] Bahga A., Madisetti V.K. (2016). Blockchain platform for industrial internet of things. J. Softw. Eng. Appl..

[33] Garzik J., Donnelly J.C. (2018). Blockchain 101: An introduction to the future. Handbook of Blockchain, Digital Finance, and Inclusion.

[34] Zelbst P.J., Green K.W., Sower V.E., Reyes P.M. (2012). Impact of RFID on manufacturing effectiveness and efficiency. Int. J. Oper. Prod. Manag..

[35] Kevan T. (2004). Theft and terror threats push sensors into supply chain. Front. Solut..

[36] Visich J.K., Li S., Khumawala B.M., Reyes P.M. (2009). Empirical evidence of RFID impacts on supply chain performance. Int. J. Oper. Prod. Manag..

[37] Fu Q., Zhu K. (2010). Endogenous information acquisition in supply chain management. Eur. J. Oper. Res..

[38] Borgia E. (2014). The Internet of Things vision: Key features, applications and open issues. Comput. Commun..

[39] Huh S., Cho S., Kim S. Managing IoT devices using blockchain platform. Proceedings of the 2017 19th International Conference on Advanced Communication Technology (ICACT).

[40] Kumar N.M., Mallick P.K. (2018). Blockchain technology for security issues and challenges in IoT. Procedia Comput. Sci..

[41] Kamran M., Khan H.U., Nisar W., Farooq M., Rehman S.U. (2020). Blockchain and Internet of Things: A bibliometric study. Comput. Electr. Eng..

[42] Fernández-Caramés T.M., Fraga-Lamas P. (2018). A Review on the Use of Blockchain for the Internet of Things. IEEE Access.

[43] Hepp T., Schoenhals A., Gondek C., Gipp B. (2018). OriginStamp: A blockchain-backed system for decentralized trusted timestamping. It-Inf. Technol..

[44] Schweizer A., Knoll P., Urbach N., von der Gracht H.A., Hardjono T. (2020). To what extent will blockchain drive the machine economy? Perspectives from a prospective study. IEEE Trans. Eng. Manag..

[45] Farooque M., Jain V., Zhang A., Li Z. (2020). Fuzzy DEMATEL analysis of barriers to Blockchain-based life cycle assessment in China. Comput. Ind. Eng..

[46] Linstone H.A., Turoff M. (1975). The Delphi Method Techniques and Applications.

[47] Dalkey N., Helmer O. (1963). An experimental application of the Delphi method to the use of experts. Manag. Sci..

[48] Bendavid Y., Cassivi L. (2010). Bridging the gap between RFID/EPC concepts, technological requirements and supply chain e-business processes. J. Theor. Appl. Electron. Commer. Res..

[49] Subramanian H. (2017). Decentralized blockchain-based electronic marketplaces. Commun. ACM.

[50] Atzori L., Iera A., Morabito G. (2010). The internet of things: A survey. Comput. Netw..

[51] Levine M. (2017). Cargo Blockchains and Deutsche Bank. https://www.bloomberg.com/view/articles/2017-03-06/cargo-blockchains-and-deutsche-bank.

[52] Christidis K., Devetsikiotis M. (2016). Blockchains and smart contracts for the internet of things. IEEE Access.

[53] Koetsier J. (2017). Blockchain Beyond Bitcoin: How Blockchain Will Transform Business in 3–5 Years. Mobile Economist.

[54] Francisco K., Swanson D. (2018). The supply chain has no clothes: Technology adoption of blockchain for supply chain transparency. Logistics.

[55] Ma H.D. (2011). Internet of things: Objectives and scientific challenges. J. Comput. Sci. Technol..

[56] Li C., Zhang L.J. A blockchain based new secure multi-layer network model for Internet of Things. Proceedings of the 2017 IEEE International Congress on Internet of Things (ICIOT).

[57] Sharma P.K., Chen M.Y., Park J.H. (2017). A software defined fog node based distributed blockchain cloud architecture for IoT. IEEE Access.

[58] Sakakibara Y., Nakamura K., Matsutani H. An fpga nic based hardware caching for blockchain. Proceedings of the 8th International Symposium on Highly Efficient Accelerators and Reconfigurable Technologies.

[59] Lamming R.C., Caldwell N.D., Harrison D., Phillips W. (2001). Transparency in supply relationships: Concept and practice. J. Supply Chain. Manag..

